# The Expression and Prognostic Significance of Retinoic Acid Metabolising Enzymes in Colorectal Cancer

**DOI:** 10.1371/journal.pone.0090776

**Published:** 2014-03-07

**Authors:** Gordon T. Brown, Beatriz Gimenez Cash, Daniela Blihoghe, Petronella Johansson, Ayham Alnabulsi, Graeme I. Murray

**Affiliations:** 1 Pathology, Division of Applied Medicine, School of Medicine and Dentistry, University of Aberdeen, Aberdeen, United Kingdom; 2 Vertebrate Antibodies, Zoology Building, Tillydrone Avenue, Aberdeen, United Kingdom; 3 George S. Wise Faculty of Life Sciences, Department of Zoology, Tel Aviv University, Tel Aviv, Israel; 4 The Scottish Fish Immunology Research Centre, School of Biological Sciences, University of Aberdeen, Aberdeen, United Kingdom; University of Kentucky College of Medicine, United States of America

## Abstract

Colorectal cancer is one of the most common types of cancer with over fifty percent of patients presenting at an advanced stage. Retinoic acid is a metabolite of vitamin A and is essential for normal cell growth and aberrant retinoic acid metabolism is implicated in tumourigenesis. This study has profiled the expression of retinoic acid metabolising enzymes using a well characterised colorectal cancer tissue microarray containing 650 primary colorectal cancers, 285 lymph node metastasis and 50 normal colonic mucosal samples. Immunohistochemistry was performed on the tissue microarray using monoclonal antibodies which we have developed to the retinoic acid metabolising enzymes CYP26A1, CYP26B1, CYP26C1 and lecithin retinol acyl transferase (LRAT) using a semi-quantitative scoring scheme to assess expression. Moderate or strong expression of CYP26A1was observed in 32.5% of cancers compared to 10% of normal colonic epithelium samples (p<0.001). CYP26B1 was moderately or strongly expressed in 25.2% of tumours and was significantly less expressed in normal colonic epithelium (p<0.001). CYP26C1 was not expressed in any sample. LRAT also showed significantly increased expression in primary colorectal cancers compared with normal colonic epithelium (p<0.001). Strong CYP26B1 expression was significantly associated with poor prognosis (HR = 1.239, 95%CI = 1.104–1.390, χ^2^ = 15.063, p = 0.002). Strong LRAT was also associated with poorer outcome (HR = 1.321, 95%CI = 1.034–1.688, χ^2^ = 5.039, p = 0.025). In mismatch repair proficient tumours strong CYP26B1 (HR = 1.330, 95%CI = 1.173–1.509, χ^2^ = 21.493, p<0.001) and strong LRAT (HR = 1.464, 95%CI = 1.110–1.930, χ^2^ = 7.425, p = 0.006) were also associated with poorer prognosis. This study has shown that the retinoic acid metabolising enzymes CYP26A1, CYP26B1 and LRAT are significantly overexpressed in colorectal cancer and that CYP26B1 and LRAT are significantly associated with prognosis both in the total cohort and in those tumours which are mismatch repair proficient. CYP26B1 was independently prognostic in a multivariate model both in the whole patient cohort (HR = 1.177, 95%CI = 1.020–1.216, p = 0.026) and in mismatch repair proficient tumours (HR = 1.255, 95%CI = 1.073–1.467, p = 0.004).

## Introduction

Colorectal cancer is one of the commonest types of malignancy whose 5 year survival remains at approximately fifty percent despite the introduction of bowel cancer screening programmes [1]. While the molecular pathogenesis of this type of tumour is increasingly being understood and defined especially the early stages of colorectal cancer development where the molecular changes have been delineated with a high degree of detail [2]–[4]. However, there is still a clear need to identify biomarkers of colorectal cancer including prognostic, predictive and diagnostic markers [5]–[15].

Retinoic acid (RA) is a metabolite of vitamin A (retinol), which performs essential functions in normal cell growth and differentiation and dysregulated retinoic acid metabolism has been implicated in tumourigenesis [16], [17]. Retinoids, a term used to describe natural or synthetic compounds showing a structural or functional resemblance to retinol, have prominent roles to play in cell growth, differentiation and apoptosis [16]. The most active form of RA, all-trans retinoic acid (atRA), has a gene regulatory function and plays a crucial role in development of the multiple organs. 4-oxo-9-cis-retinoic acid (9-cis-RA) and 4-oxo-13-cis-retinoic acid (13-cis-RA) are stereo-isomers of atRA and also play an important role in RA signalling. Some retinoids possess anti-cancer properties that have already been exploited for the treatment of several types of cancer including cervical cancer and promyelocytic leukaemia.

The intracellular processing of retinol involves lecithin retinol acyl transferase (LRAT) which is responsible for the esterification of retinol [18], [19] while hydroxylation of retinol is performed by the retinoic acid hydroxylases (CYP26A1, CYP26B1, CYP26C1) which are all members of the cytochrome P450 (P450) family of enzymes [20], [21].

The three members of the CYP26 family are all capable of metabolising atRA into less biologically active 4-hydroxy-, 4-oxo-, and 18-hydroxy-RA intermediates [22]–[24], of which, 4-oxo-RA is the most common metabolite [16]. Although previous studies have investigated P450 expression in tumours and shown tumour selective expression of individual P450s most notably CYP1B1 [25] the CYP26 family of P450s has received little prior attention in relation to their expression in tumours.

This study has profiled the expression of the retinoic acid metabolising enzymes CYP26A1, CYP26B1, CYP26C1 and LRAT using a well characterised colorectal cancer tissue microarray with monoclonal antibodies to CYP26A1, CYP26B1, CYP26C1 and LRAT respectively, that have been developed and characterised for their use by immunohistochemistry on formalin fixed wax embedded tissue.

## Materials and Methods

### Monoclonal antibodies

Monoclonal antibodies to CYP26A1, CYP26B1, CYP26C1 and LRAT were developed in collaboration with Vertebrate Antibodies Ltd (Aberdeen, UK) using synthetic peptides. Peptides within the putative protein sequences were identified which were antigenic, exposed on the surface and unique to the target protein. The amino acid sequences and location on the proteins are indicated in table 1. The peptides were obtained from Almac Sciences Ltd, (Edinburgh, UK) and conjugated individually to ovalbumin for the immunisations and to bovine serum albumin for the ELISA tests [26]. The immunisation of mice, production of hybridoma cells and ELISA screening were carried out essentially as described previously [26] except that the antigen was given by subcutaneous route. The hybridomas were cloned by limiting dilution until a single ELISA positive colony was grown in a 96 well plate. Individual cell lines were then grown at high cell density for the preparation of the antibody stock which was used subsequently for their characterisation by immunoblotting and immunohistochemistry.

**Table 1:** Details of amino acid sequences used as peptide immunogens.

Enzyme	Amino acid sequence	Location of peptide
CYP26A1	PARFTHFHGE	487–496
CYP26B1	DSNQNEILPE	494–503
CYP26C1	RWELATPAFP	481–490
LRAT	RDQRSVLASA	190–199

### Immunoblotting

Whole cell lysates from cells (human embryonic kidney cells) overexpressing CYP26A1, CYP26B1, CYP26C1 and LRAT were used as positive controls for immunoblotting while lysates from cells containing vector only were used as negative controls. The CYP26A1 cell lysate and its control were purchased from Abnova (Taipei, Taiwan) while CYP26B1, CYP26C1 and LRAT containing cell lysates and their corresponding controls were obtained from (Novus Biologicals, Cambridge, UK). Cell lysates (5 µg protein/lane) were resolved by electrophoresis on NuPAGE 4-12% bis-Tris gels (Fisher Scientific, Loughborough, UK). Following protein transfer the membranes were blocked for 1 hour at room temperature in phosphate buffered saline-Tween-20 (PBST) containing 1% (w/v) skimmed milk powder. Membranes were incubated overnight at 4°C with CYP26A1 (1/5 dilution), CYP26B1 (1/2 dilution), CYP26C1 (1/2 dilution) or LRAT (1/2 dilution) monoclonal antibodies diluted in PBST. Membranes were washed (6 times) for 1 hour in 1% skimmed milk. The membranes were subsequently probed for 1 hour with a secondary antibody conjugated horseradish-peroxidase-conjugated anti-mouse IgG (1/2000, Sigma-Aldrich, Dorset, UK). Membranes were then washed (6 times) for 1 hour in 1% skimmed milk and protein bands visualized using the enhanced chemiluminescence detection system (Fisher Scientific).

### Colorectal cancer tissue microarray

All cases of colorectal cancer included in this study were retrospectively selected from the Aberdeen colorectal tumour bank (now incorporated in and governanced by the NHS Grampian Biorepository, Aberdeen, UK). In total, tumour samples from 650 patients were involved in this study, in each case, a diagnosis of primary colorectal cancer had been made, and the patients had undergone elective surgery for primary colorectal cancer, in Aberdeen, between 1994 and 2009. None of the patients had received any form of pre-operative chemotherapy or radiotherapy. In particular patients with rectal cancer who had received neoadjuvant therapy including short course radiotherapy were excluded. The data for the patients and their tumours included in this study is detailed in Table S1. Survival information (all cause mortality) was available for all patients and at the time of censoring patient outcome data there had been 309 (47.5%) deaths. The mean patient survival was 115 months (95% CI 108–123 months).

The tumours were reported according to The Royal College of Pathologists UK guidelines for the histopathological reporting of colorectal cancer resection specimens and which incorporates guidance from version 5 of the TNM staging system [27]. The histopathological processing of the colorectal cancer excision specimens is detailed in Materials and Methods S1.

A colorectal cancer tissue microarray was constructed containing normal colon mucosal samples (n = 50), primary (n = 650) and metastatic colorectal cancer samples (n = 285) as previously described [28]–[30]. Details of the construction of the tissue microarray are given in Materials and Methods S1.

### Immunohistochemistry

Immunohistochemistry for each antibody was performed with the biotin-free Dako Envision system (Dako, Ely, UK) using a Dako autostainer (Dako) as previously described [28]–[30]. Details of the immunohistochemistry are given in Materials and Methods S1. The sections were evaluated by light microscopic examination and the intensity of immunostaining in each core assessed independently by two investigators (GTB and GIM) using a scoring system previously described for the assessment of protein expression in tumour microarrays [28]–[30]. The intensity of immunostaining in each core was scored as negative, weak, moderate or strong. The sub-cellular localisation (nuclear, cytoplasmic or membranous) of the immunostaining was also recorded. Variation in immunostaining between cores of each case was not identified. Any discrepancies in the immunohistochemical assessment of the tissue cores between the two observers were resolved by simultaneous microscopic re-evaluation.

### Assessment of mismatch repair status

Mismatch repair status (MMR) was assessed by immunohistochemistry using antibodies to MLH1 and MSH2 as described previously [28], [30]. MMR status was recorded as either proficient or defective.

### Statistics

Statistical analysis of the data including the Mann-Whitney U test, Wilcoxon signed rank test, chi-squared test, Kaplan-Meier survival analysis, log-rank test and Cox multi-variate analysis (variables entered as categorical variables) including the calculation of hazard ratios and 95% CIs was performed using IBM SPSS version 21 for Windows 7™ (IBM, Portsmouth, UK). The log rank test was used to determine survival differences between individual groups. A probability value of p≤0.05 was regarded as significant. The influence of different cut-off points in relation to survival was investigated by dichotomizing the intensity score for each marker. The groups that were analysed were negative staining versus any positive staining, negative and weak staining versus moderate and strong staining and negative, weak and moderate staining versus strong staining.

### Ethics

The project had the approval of The North of Scotland research ethics committee (ref. nos. 08/S0801/81 and 11/NS/0015). The research ethics committee did not require written patient consent for the retrospective tissue samples that were included in the colorectal cancer tissue microarray.

## Results

### Monoclonal antibodies

**Figure 1:**
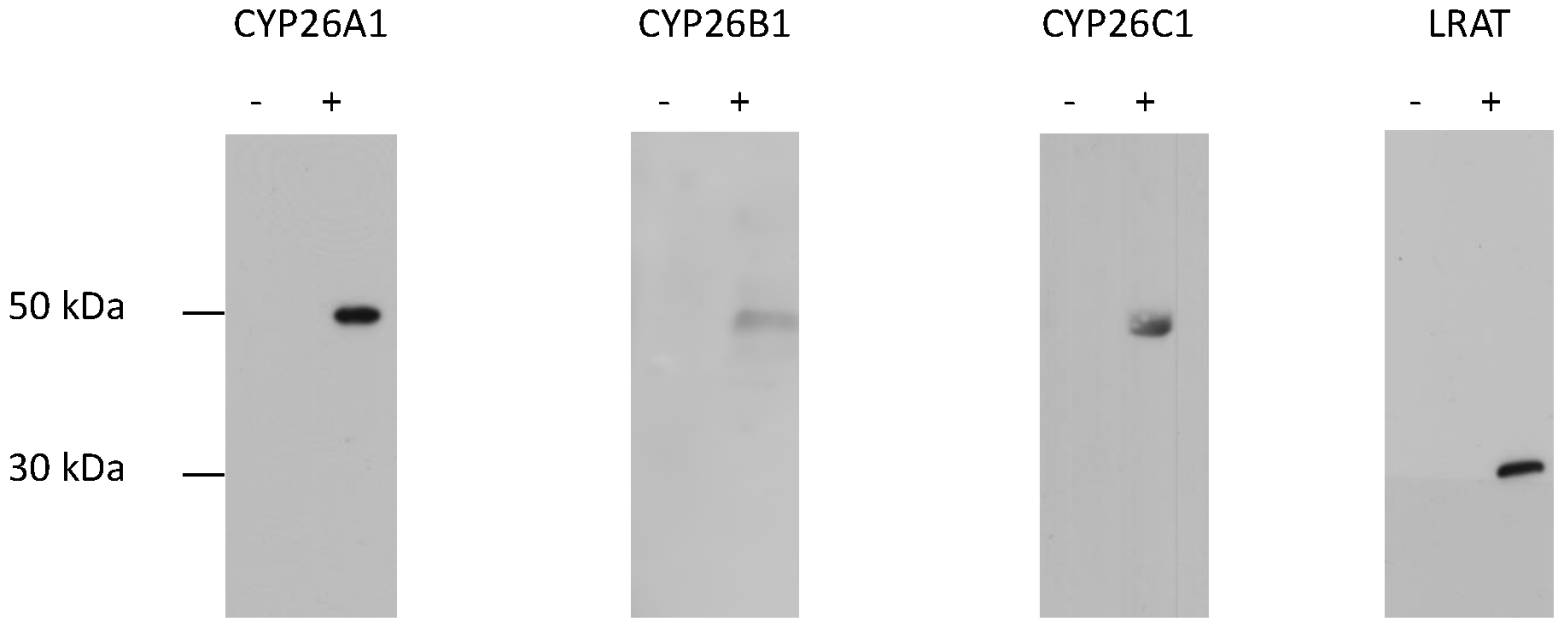
Immunoblots of CYP26A1, CYP26B1, CYP26C1 and LRAT antibodies. The left hand lane of each panel contains control cell lysate while the right hand lane of each panel contains lysate prepared from cells over expressing the relevant protein. 5 micrograms of protein were loaded per well.

The specificity of the monoclonal antibodies to CYP26A1, CYP26B1, CYP26C1 and LRAT were determined by ELISA using the immunogenic peptides and also by immunoblotting (figure 1) using whole cell lysates from cells overexpressing each protein. A band migrating at the expected molecular weight was observed for each antibody in a lysate prepared from cells overexpressing the relevant protein while no bands were detected with the corresponding control lysate.

### Immunohistochemistry

**Figure 2:**
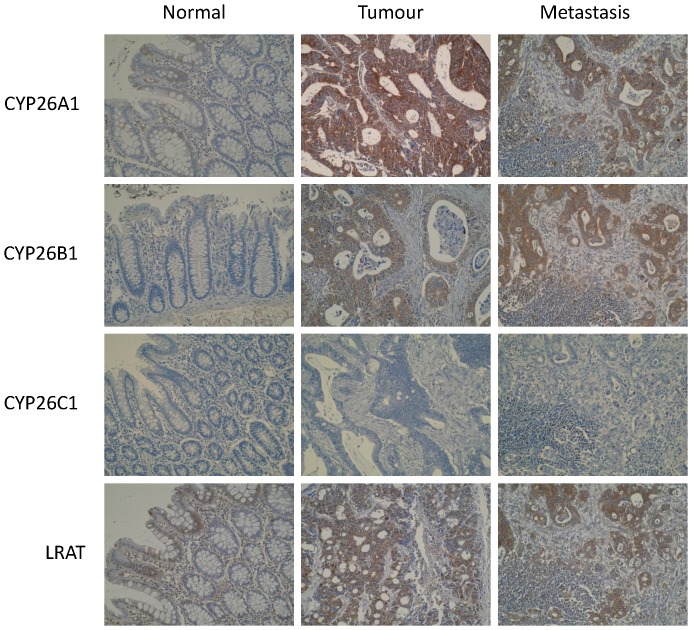
Photomicrographs of CYP26A1, CYP26B1, CYP26C1 and LRAT in normal colonic mucosa, primary colorectal cancer and metastatic colorectal cancer.

CYP26A1, CYP26B1 and LRAT all showed immunoreactivity in both normal colonic epithelium and primary and metastatic colorectal cancer and in each case immunostaining was localised to the cytoplasm of cells (figure 2). There was no nuclear or cell surface membrane imunoreactivity. In normal colonic epithelium immunostaining was predominantly localised surface epithelial cells and no intratumour heterogeneity was observed in either primary or metastatic colorectal tumours. CYP26C1 did not show any immunostaining in any of the tissue samples examined.

**Figure 3:**
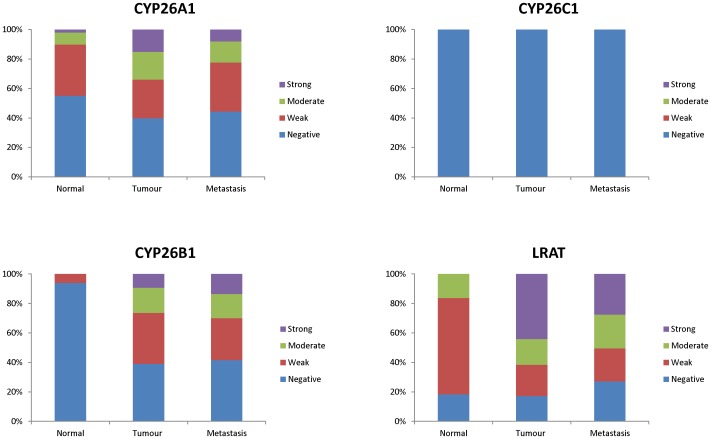
The frequency distribution of the intensity of expression of CYP26A1, CYP26B1, CYP26C1 and LRAT in normal colonic mucosa, primary colorectal cancer and metastatic colorectal cancer.

The intensity of immunostaining was significantly higher in primary colorectal cancer compared with normal colonic mucosa for CYP26A1 (p = 0.002), CYP26B1 (p<0.001) and LRAT (p<0.001) (figure 3, table 2). There was no difference in the intensity of expression of either CYP26A1 or CYP26B1 between Dukes C colorectal cancer and their corresponding lymph node metastasis whereas for LRAT there was a significant decrease in immunoreactivity in the lymph node metastasis compared with the corresponding primary tumours (p<0.001) (figure 3 and table 2).

**Table 2:** Comparison of CYP26A1, CYP26B1 and LRAT expression in normal colonic mucosa, primary colorectal cancer and lymph node metastasis.

	Immunoreactivity (p value, normal versus primary tumour)	Change in expression in tumour	Immunoreactivity (p value, primary tumour versus lymph node metastasis)	Change in expression in lymph node	Immunoreactivity (p value, paired primary Dukes C tumour versus lymph node metastasis)	Change in expression in lymph node
CYP26A1	**0.002**	↑	**0.015**	↓	0.208	↔
CYP26B1	**<0.001**	↑	0.822	↔	0.656	↔
CYP26C1	-	-	-	-	-	-
LRAT	**<0.001**	↑	**<0.001**	↓	**<0.001**	↓

Evaluation of normal colonic epithelium versus primary tumour samples for immunoreactivity (Mann-Whitney U test, ↑ = increased in tumour, ↓ = decreased in tumour, - = no change between tumour and normal) and evaluation of primary Dukes C colorectal tumour samples and their corresponding metastasis samples for immunoreactivity (Wilcoxon signed rank sum test, ↑ = increased in lymph node metastasis, ↓ = decreased in lymph node metastasis, ↔ = no change between primary and metastatic tumour). Significant values are highlighted in bold.

The expression of CYP26A1 was strongly correlated with both CYP26B1 expression (χ^2^ = 192.2, p<0.001) and LRAT expression (χ^2^ = 89.54, p<0.001) while CYP26B1 expression also correlated with the expression of LRAT (χ^2^ = 144.88, p<0.001).

### Relationship with clinico-pathological parameters

Comparisons of the expression of CYP26A1, CYP26B1 and LRAT and clinico-pathological parameters are summarised in table 3. CYP26B1 and LRAT both showed significant associations with tumour site, tumour (T) stage, extramural venous invasion and overall stage. In contrast, CYP26A1 only showed a relationship with tumour site and did not show a relationship with any of the other clinico-pathological parameters investigated.

**Table 3:** The relationship of CYP26A1, CYP26B1 and LRAT with pathological parameters.

	Tumour site		Tumour differentiation		pT stage		pN stage		Dukes stage		EMVI		MMR status		Bowel screen detected	
	χ^2^	p value	χ^2^	p value	χ^2^	p value	χ^2^	p value	χ^2^	p value	χ^2^	p value	χ^2^	p value	χ^2^	p value
CYP26A1	13.000	**0.043**	1.625	0.654	15.728	0.073	9.476	0.136	11.545	0.073	3.03	0.387	0.613	0.894	1.367	0.713
CYP26B1	25.723	**0.002**	1.760	0.624	25.723	**0.002**	10.743	0.097	21.000	**0.002**	8.839	**0.032**	1.649	0.648	5.192	0.158
CYP26C1	-	-	-	-	-	-	-	-	-	-	-		-	-	-	-
LRAT	29.861	**<0.001**	3.815	0.282	29.861	**<0.001**	3.073	0.800	22.208	**0.001**	8.563	**0.036**	1.198	0.754	5.624	0.131

Significant values are highlighted in bold.

### Survival analysis

#### Whole patient cohort

**Figure 4:**
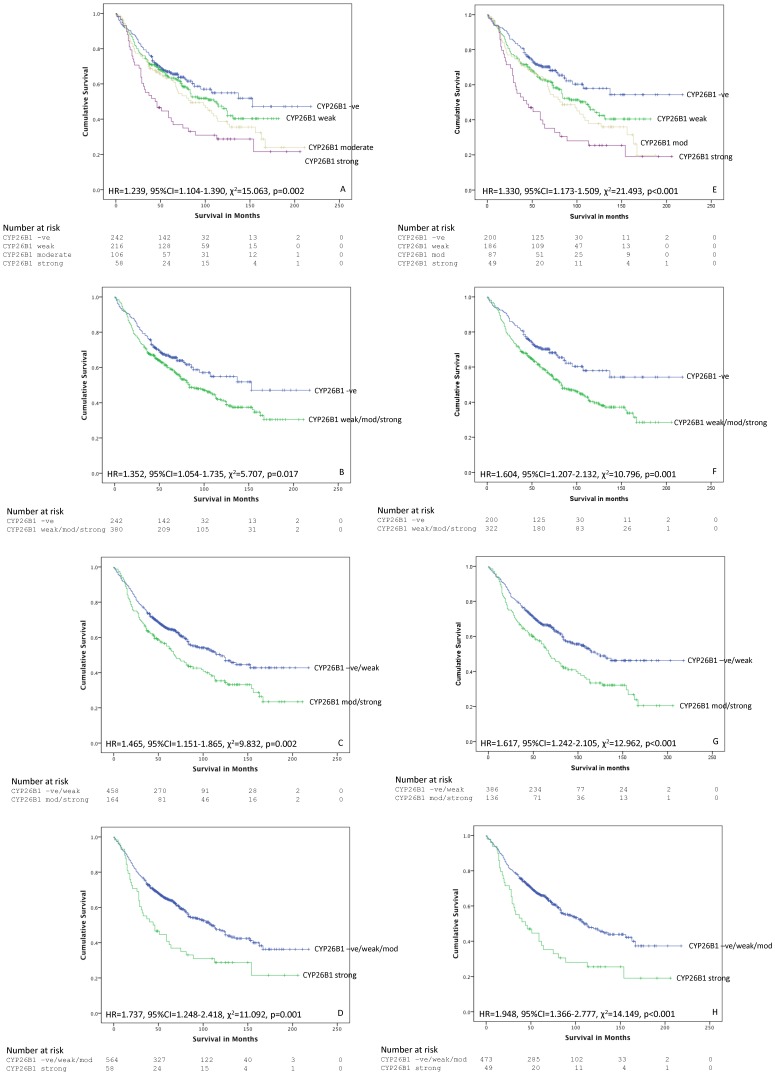
The relationship of CYP26B1 and survival. A–D whole patient cohort. A. overall survival showing individual CYP26B1 categories. In B–D the influence of different cut of points is shown. E–H Mismatch repair proficient cohort. E. overall survival showing individual CYP26B1 categories. In F–H the influence of different cut-off points is shown.

The relationship of the expression of CYP26A1, CYP26B1 and LRAT with overall survival was investigated using different cut-off points (negative v weak v moderate v strong, negative v positive, negative/weak positive v moderate/strong and negative/weak/moderate v strong) of the immunohistochemical scoring and is summarised in table 4 and figure 4.

**Table 4:** The relationship of CYP26A1, CYP26B1 and LRAT expression with survival (log rank test) using different cut-off points for the immunohistochemical data.

	Negative versus weak versus moderate versus strong		Negative versus weak and moderate and strong		Negative and weak versus moderate and strong		Negative, weak and moderate versus strong	
	χ^2^	p value	χ^2^	p value	χ^2^	p value	χ^2^	p value
CYP26A1	0.701	0.873	0.487	0.485	0.032	0.858	0.165	0.685
CYP26B1	15.063	**0.002**	5.707	**0.017**	9.832	**0.002**	11.092	**0.001**
CYP26C1	-	-	-	-	-	-	-	-
LRAT	5.329	0.149	2.485	0.115	5.039	**0.025**	3.489	0.062

Significant values are highlighted in bold.

There was a consistent and significant association between CYP26B1 expression and outcome (Figure 4). Considering each CYP26B1 intensity group separately, increasing intensity of CYP26B1 immunoreactivity was associated with poorer prognosis (HR = 1.239, 95%CI = 1.104–1.390, χ^2^ = 15.063, p = 0.002). For CYP26B1 negative tumours (n = 242) the mean survival was 133 months (95%CI = 118–148 months), for CYP26B1 weak expressing tumours (n = 216) the mean survival was 106 months (95%CI = 95–116 months), for CYP26B1 moderate expressing tumours (n = 106) the mean survival was 103 months (95%CI = 85–120 months) and for strongly expressing CYP26B1 tumours (n = 58) the mean survival was 81 months (95%CI = 60–101 months).

Comparing CYP26B1 negative tumours with tumours that showed any CYP26B1 immunoreactivity then poorer survival was associated with CYP26B1 expression (HR = 1.352, 95%CI = 1.054–1.735, χ^2^ = 5.707, p = 0.017). For CYP26B1 negative tumours (n = 242) the mean survival was 133 months (95%CI = 118–148 months) while for CYP26B1 positive tumours (n = 380) the mean survival was 107 months (95%CI = 98–116 months). Comparing CYP26B1 negative and weakly positive tumours with CYP26B1 moderate and strong expressing tumours showed that there was a highly significant association with survival (HR = 1.465, 95%CI = 1.151–1.865, χ^2^ = 9.832, p = 0.002). Mean survival for the negative/weak tumours (n = 458) was 125 months (95%CI = 115–135 months) while the mean survival for the moderate/strong group of tumours (n = 164) was 96 months (95%CI = 108–124 months). CYP26B1 negative/weak/moderate expressing tumours when compared with CYP26B1 strongly expressing tumours also showed a highly significant association with survival (HR = 1.737, 95%CI = 1.248–2.418, χ^2^ = 11.092, p = 0.001). The mean survival for the CYP26B1 negative/weak/moderate group (n = 564) was 119 months (95%CI = 111–128 months) whereas the mean survival for CYP26B1 strong tumours (n = 58) was 80 months (95%CI = 60–101 months).

**Figure 5:**
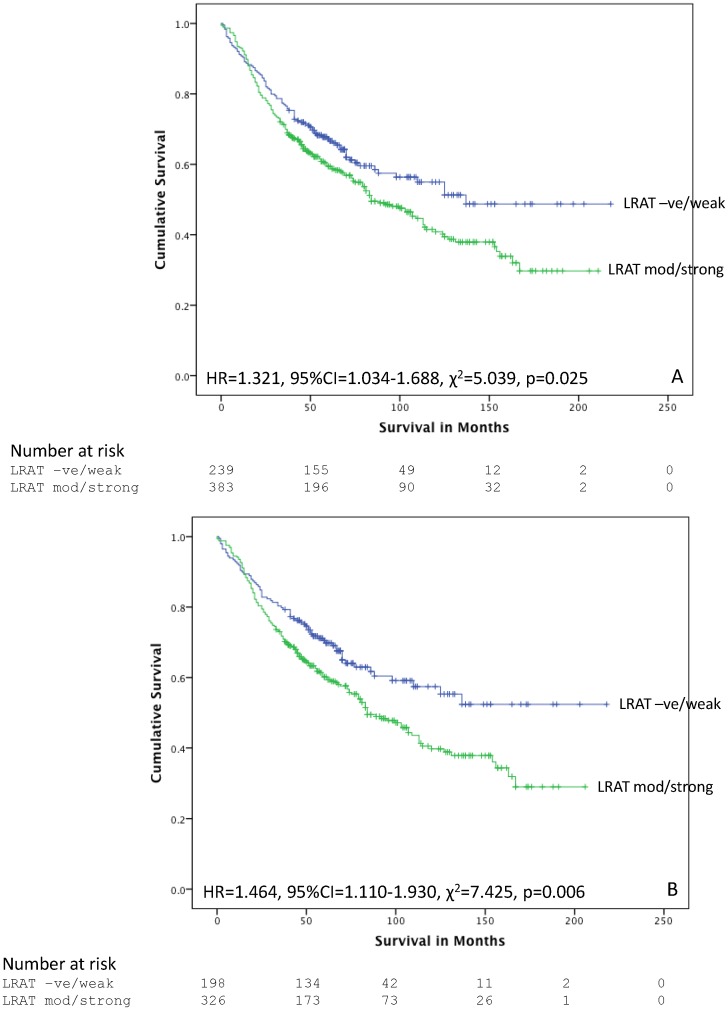
The relationship of LRAT and survival. **A.** The relationship of LRAT and survival in all colorectal cancers and **B.** The relationship of LRAT and survival in mismatch repair proficient tumours.

For LRAT there was a significant relationship with survival (HR = 1.321, 95%CI = 1.034–1.688, χ^2^ = 5.039, p = 0.025) when the immunohistochemistry intensity score were dichotomised into LRAT negative and weak cases (n = 239) versus LRAT moderate and strong cases (n = 383) (figure 5). For LRAT negative and weak cases mean survival was 132 months (95%CI = 119–146 months) while for LRAT moderate and strong cases mean survival was 106 months (95%CI = 96–116 months). There was no association between CYP26A1 and survival in the whole patient cohort.

The detailed relationship between CYP26A1, CYP26B1 and LRAT expression, pathological parameters and overall survival is shown in Tables S2, S3, S4, and S5.

In multivariate analysis CYP26B1 remained independently prognostic (p = 0.026) while there was no independent prognostic significance of LRAT expression (table 5). If the multivariate analysis model contains only the variables that would be available from a biopsy of colorectal cancer (i.e. no information regarding pT stage, pN stage and EMVI) then CYP26B1 is a significant independent prognostic marker (p = 0.017, Table S6)

**Table 5:** Multivariate analysis of whole patient cohort.

Variable	Wald value	p-value	Hazard ratio (95%CI)
Age	25.027	**<0.001**	1.899 (1.477–2.442)
Gender	1.313	0.252	0.871 (0.687–1.103)
Tumour site	6.390	**0.041**	0.983 (0.713–1.909)
Tumour differentiation	4.469	**0.035**	0.603 (0.377–0.964)
Tumour (pT) stage	21.910	**<0.001**	0.486 (0.234–1.010)
Nodal (pN) stage	68.015	**<0.001**	0.255 (0.184–0.702)
EMVI	20.064	**<0.001**	1.872 (1.423–2.463)
MMR status	0.245	0.620	0.921 (0.666–1.274)
CYP26B1	4.962	**0.026**	1.177 (1.020–1.216)
LRAT	0.482	0.487	0.663–1.216)

#### MMR proficient cohort

In MMR proficient tumours there was a consistent relationship between the intensity of CYP26B1 expression and overall survival (table 6, figure 4). Increasing intensity of CYP26B1 immunoreactivity was associated with poorer prognosis (HR = 1.330, 95%CI = 1.173–1.509, χ^2^ = 21.493, p<0.001). For CYP26B1 negative tumours (n = 200) the mean survival was 143 months (95%CI = 127–158 months), for CYP26B1 weak tumours (n = 186) the mean survival was 106 months (95%CI = 94–116 months), for CYP26B1 moderate tumours (n = 87) the mean survival was 96 months (95%CI = 82–112 months) and for strongly expressing CYP26B1 tumours (n = 49) the mean survival was 77 months (95%CI 56–98 months).

**Table 6:** The relationship of the expression of CYP26A1, CYP26B1 or LRAT and survival in MMR proficient and defective colorectal cancers.

			Negative versus weak versus moderate versus strong		Negative versus weak/moderate/strong		Negative/weak versus moderate/strong		Negative/weak/moderate versus strong	
			χ^2^	p-value	χ^2^	p-value	χ^2^	p-value	χ^2^	p-value
CYP26A1										
	MMR Status									
		Proficient	2.475	0.480	1.184	0.276	0.983	0.321	2.033	0.154
		Defective	4.123	0.248	0.693	0.405	2.902	0.088	3.435	0.064
CYP26B1										
	MMR Status									
		Proficient	21.493	**<0.001**	10.869	**0.001**	13.050	**<0.001**	14.149	**<0.001**
		Defective	1.809	0.613	1.669	0.196	0.130	0.718	<0.001	0.992
LRAT										
	MMR Status									
		Proficient	8.432	**0.038**	5.076	**0.024**	7.425	**0.006**	5.199	**0.023**
		Defective	0.486	0.962	0.407	0.523	0.179	0.672	0.224	0.636

Significant values are highlighted in bold.

Comparing CYP26B1 negative tumours with tumours that showed any CYP26B1 immunoreactivity then poorer survival was associated with CYP26B1 expression (HR = 1.604, 95%CI = 1.207–2.132, χ^2^ = 10.796, p = 0.001). For CYP26B1 negative tumours (n = 200) the mean survival was 143 months (95%CI = 127–158 months) while for CYP26B1 positive tumours (n = 322) the mean survival was 104 months (95%CI = 94–114 months). Comparing CYP26B1 negative and weakly positive tumours with CYP26B1 moderate and strong expressing tumours showed a highly significant association with survival (HR = 1.617, 95%CI = 1.242–2.105, χ^2^ = 12.962, p<0.001). Mean survival for the negative/weak tumours (n = 386) was 130 months (95%CI = 120–141 months) and the mean survival for the moderate/strong group of tumours was 92 months (95%CI = 79–106 months). CYP26B1 negative/weak/moderate expressing tumours when compared with CYP26B1 strongly expressing tumours also showed a highly significant association with survival (HR = 1.948, 95%CI = 1.366–2.777, χ^2^ = 14.149, p<0.001). The mean survival for the CYP26B1 negative/weak/moderate group (n = 473) was 123 months (95%CI = 113–132 months) and the mean survival for CYP26B1 strongly expressing tumours was 77 months (95%CI = 56–98 months).

For LRAT there was a significant relationship with survival (HR = 1.464, 95%CI = 1.110–1.930, χ^2^ = 7.425, p = 0.006) when the immunohistochemistry intensity scores were dichotomised into LRAT negative and weak cases (n = 198) versus LRAT moderate and strong cases (n = 326) (figure 5). For LRAT negative and weak cases mean survival was 139 months (95%CI = 124–156 months) while for LRAT moderate and strong cases mean survival was 106 months (95%CI = 96–116 months). There was no association between CYP26A1 and survival in the MMR proficient patient cohort.

In multivariate analysis CYP26B1 remained independently prognostic (p = 0.026) while there was no independent prognostic significance of LRAT expression (table 7). If the multivariate analysis model contains only the variables that would be available from a biopsy of colorectal cancer (i.e. no information regarding tumour stage, nodal stage and extra-mural venous invasion) then CYP26B1 is a highly significant independent prognostic marker (p = 0.001, Table S6)

**Table 7:** Multivariate analysis of MMR proficient cases.

Variable	Wald value	p-value	Hazard ratio (95%CI)
Age	26.009	**<0.001**	2.045 (1.553–2.692)
Gender	3.381	0.068	0.782 (0.601–1.016)
Tumour site	4.108	0.128	0.946 (0.677–1.836)
Tumour differentiation	7.941	**0.005**	0.420 (0.230–0.768)
Tumour (pT) stage	15.314	**0.002**	0.510 (0.241–1.080)
Nodal (pN) stage	49.405	**<0.001**	0.263 (0.181–0.690)
EMVI	14.120	**<0.001**	1.808 (1.327–2.461)
CYP26B1	8.091	**0.004**	1.255 (1.073–1.467)
LRAT	1.969	0.161	0.787 (0.563–1.100)

Significant values are highlighted in bold.

There was no relationship of MMR defective tumours with CYP26A1, CYP26B1 or LRAT expression and overall survival.

## Discussion

Colorectal cancer is one of the commonest types of cancer whose incidence is continuing to rise and while the molecular events characterising the early stage of colorectal cancer development have been described in detail there is still clear requirement to identify, characterise and validate biomarkers of colorectal cancer [4]–[6], [12].

This study has defined the expression profile of the retinoic acid metabolising enzymes CYP26A1, CYP26B1 and CYP26C1 which are members of the P450 family of enzymes and LRAT in a large cohort of well characterised colorectal cancers. The prognostic significance of the expression of these retinoic acid metabolising enzymes has also been established.

The P450s are a large group of NADP dependent hydroxylases classified into families, sub-families and individual members [31]–[34]. There are two distinct functional groups of P450s based on their substrate specificity for either xenobiotics or endogenous compounds. CYP1, CYP2 and CYP3 are the predominant families involved in the metabolism of xenobiotics while other families from CYP4 upwards are involved in the metabolism of specific groups of biologically active endogenous compounds including eicosanoids, steroid hormones, bile acids and vitamins including vitamin A and D [35]–[42]. They have multiple transcriptional and post-transcriptional regulatory mechanisms [43]. The xenobiotic metabolising forms of P450 have been extensively studied in tumours and many individual members have been shown to be overexpressed in specific types of tumours most notably CYP1B1 which has been shown to have increased expression in a wide range of tumours [25], [44]–[52]. The tumour selective expression of P450 has been proposed as a therapeutic target especially for P450 mediated pro-drug activation [51]–[57]. The P450s involved in endogenous compound metabolism have generally received much less study in tumours with the exception of those P450s involved in sex hormone (oestrogen and testosterone) metabolism in relation to targets in breast and prostate cancer respectively [58]–[60].

Structurally the P450s show greatest amino acid diversity at their C-termini which is the hydrophilic component of the protein in contrast to the N-terminus which is the most lipophilic component of the protein. Given the marked C-terminal amino acid variation and its hydrophilicity the use of peptides to the C-terminus of individual P450 as immunogens to produce monoclonal antibodies to individual forms of P450 has proven for many research groups including our own to be a highly efficient strategy to develop individual form-specific P450 monoclonal and polyclonal antibodies [30], [61], [62].

In this study we have produced antibodies that are specific for individual forms of the CYP26 family namely CYP26A1, CYP26B1 and CYP26C1. All three CYP26 enzymes hydroxylate retinoic acid and the most fully characterised is CYP26A1 which is the predominant form involved in retinoic acid hydroxylation. CYP26B1 and CYP26C1 are more recently identified members of the CYP26 family and are less well characterised in comparison with CYP26A1. CYP26C1 appears to have predominant but not necessarily exclusive expression in specific regions of the brain [22], [23].

Colorectal cancers can be classified according to their microsatellite instability/MMR status and this represents a major pathway of colorectal cancer development [63], [64]. In this study we found prognostic significance for CYP26B1 in both the whole patient cohort and in those tumours which were defined as microsatellite intact or stable. In contrast those tumours which were MMR defective did not show any prognostic significance suggesting that distinct regulatory mechanisms may be operating in MMR proficient and MMR defective tumours.

The CYP26 enzymes have been proposed as anti-cancer drug targets [65] and the increased expression of CYP26A1 and CYP26B1 in colorectal cancer would suggest that these enzymes may be relevant therapeutic targets in this type of tumours. Several series of compounds based on different structural properties have been synthesised that inhibit CYP26 [66]–[69]. These compounds are highly selective for CYP26 and show high inhibitory activity (nanomolar potency) towards CYP26A1. However, the inhibitory activity of these compounds towards CYP26B1 and CYP26C1 has not yet been evaluated. Epidemiological evidence has also proposed targeting of retinoids and the retinoic acid pathways for chemoprevention of colorectal cancer and the increased expression of CYP26A1 and CYP26B1 in colorectal cancer would indicate that targeting these enzymes may be a useful approach [70]–[72].

This study also found increased expression of LRAT in primary colorectal cancer compared with normal colonic epithelium. This finding appears to contrast with previous studies of other tumour types including bladder cancer [73], breast cancer [74] and prostate cancer [75] which have suggested reduced LRAT expression in cancer cells albeit in those studies relatively small numbers of tumour samples were analysed and mainly biochemical assays of whole tumour extracts were used resulting in the assessment of an “average level” as tumour stroma and necrotic tissue will have been included. There were significant associations between LRAT expression in both the whole patient cohort and MMR proficient tumours when the LRAT scores were dichotomised into negative/weak/moderate and strong. This association was not as marked for other cut-off points and was less robust than observed for CYP26B1 in terms of prognostic significances.

**Figure 6:**
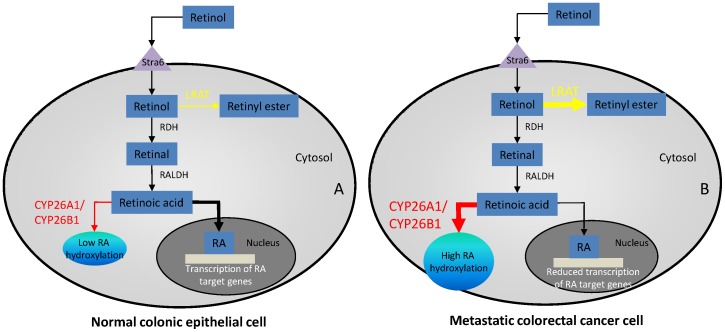
A schematic pathway indicating the interaction of retinoic acid metabolising enzymes in normal and metastatic colorectal cancer cells. A. CYP26A1, CYP26B1 and LRAT expression are low in normal cells which result in the “correct” amount of retinoic acid and expression of retinoic acid target genes to maintain and promote normal cell growth and differentiation. B. CYP26A1, CYP26B1 and LRAT show significant overexpression in metastatic colorectal cancer cells potentially reducing retinoic acid levels and retinoic acid target gene transcription which in turn significantly alters growth, differentiation and promotes a pro-metastatic phenotype. Stra6, stimulated by retinoic acid gene 6 receptor, this cell surface receptor promotes the intracellular uptake of retinol; RDH, retinol dehydrogenase; RALDH, retinaldehyde dehydrogenase.

The major problem with most types of cancer, including colorectal cancer, is metastatic disease and treatment is usually targeted at metastasis although phenotypic assessment on which treatment decisions are often made by analysis of primary tumour specimens. In contrast to the well defined molecular events leading to the development of colorectal cancer the pathways of metastasis have received much less attention [76]. This study was designed to include the assessment of phenotypic expression in both primary tumours and their corresponding lymph node metastasis. It was found that there was no difference in expression in CYP26A1 or CYP26B1 between primary tumours and corresponding lymph node metastasis. However, LRAT showed significant decrease in expression in lymph node metastasis compared with the corresponding primary tumours. This suggests both primary tumour related factors and microenvironmental factors are involved in the regulation of expression of these enzymes in metastasis of colorectal cancer. The potential consequences of altered expression of CYP26A1, CYP26B1 and LRAT in metastatic colorectal cancer cells and their contribution to a pro-metastatic phenotype are outlined in figure 6.

In summary monoclonal antibodies to individual retinoic acid metabolising enzymes have been developed that are effective on formalin fixed wax embedded tissue and shown that the retinoic acid metabolising enzymes CYP26A1, CYP26B1 and LRAT are significantly overexpressed in colorectal cancer and that CYP26B1 and LRAT are significantly associated with prognosis both in the total patient cohort and in those tumour which are MMR proficient. CYP26B1 which was independently prognostic in a multivariate model both in the whole patient cohort and in MMR proficient tumours represents a new biomarker of colorectal cancer and CYP26B1 may represent a novel drug target for this type of tumour.

## Supporting Information

Table S1 **Clinicopathological characteristics of the patients and their tumours included in the colorectal cancer tissue microarray.**
(PDF)

Table S2 **The relationship of the expression of CYP26A1, CYP26B1 and LRAT and survival in individual Dukes stage of colorectal cancer.**
(PDF)

Table S3 **The relationship of the expression of CYP26A1, CYP26B1 and LRAT and survival in colon cancer and rectal cancer.**
(PDF)

Table S4 **The relationship of the expression of CYP26A1, CYP26B1 and LRAT and survival in proximal and distal colon cancers.**
(PDF)

Table S5 **The relationship of the expression of CYP26A1, CYP26B1 and LRAT and survival in colorectal cancers with and without EMVI.**
(PDF)

Table S6 **Multi-variate analysis of the whole patient cohort and MMR proficient cohort.**
(PDF)

Materials and Methods S1 (PDF)
