# Supplementary material for: The Expression and Prognostic Significance of Retinoic Acid Metabolising Enzymes in Colorectal Cancer
Source: PLoS One. 2014 Mar 7;9(3):e90776. doi: 10.1371/journal.pone.0090776 (PMC3946526; doi:10.1371/journal.pone.0090776)
Supplement: Table S1 — Clinicopathological characteristics of the patients and their tumours included in the colorectal cancer tissue microarray. (PDF) [file pone.0090776.s001.pdf]

Supporting information

Table S1. Clinicopathological characteristics of the patients and their tumours included in the colorectal cancer tissue microarray.

		Percent (number)	Mean survival (months, 95% CI)	Relationship with survival, hazard ratio and (95% CI)
Sex				
	Male	52% (340)	114 (104-126)	χ^2=0.027, p=0.870, HR=0.982 (0.785-1.227)
	Female	48% (310)	112 (102-123)	
Age (mean:range)		(69:24-94)		
	< 70	47% (305)	140 (129-151)	χ^2=29.213, p=<0.001, HR=1.879 (1.488-2.373)
	≥ 70	53% (345)	88 (80-96)	
Tumour site[1]				
	Colon	78% (506)	113 (105-122)	Colon *v* rectum, χ^2=0.098, p=0.754 HR=1.043 (0.801-1.357)
	Proximal	40% (261)	105 (92-117)	Proximal *v* distal, χ^2= 8.418, p=0.004 HR=0.685 (0.530-0.887)
	Distal	38% (245)	121 (109-133)	Distal *v* rectal, χ^2=0.906, p=0.341, HR=0.864 (0.640-1.168)
	Rectum	22% (144)	115 (97-133)	

Tumour differentiation				
	Well/moderate	92% (600)	116 (108-124)	χ^2=0.976, p=0.323, HR=1.223 (0.818-1.829)
	Poor	8% (50)	100 (76-124)	
Tumour stage				
	T1	4.6% (30)	139 (112-165)	T1 *v* T2, χ^2=0.382, p=0.536, HR=1.264 (0.600-2.666)
	T2	17.5% (114)	163 (147-179)	T2 *v* T3, χ^2=24.739, p=<0.001, HR=0.392 (0.267-0.575)
	T3	63.2% (411)	107 (98-116)	T3 *v* T4, χ^2=30.159, p=<0.001, HR=0.466 (0.352-0.617)
	T4	14.6% (95)	56 (43-68)	
Nodal stage				
	N0	56.2% (365)	147 (137-157)	N0 *v* N1, χ^2=54.071, p=<0.001, HR=0.384 (0.295-0.501)
	N1	27.1% (176)	87 (74-101)	N1 *v* N2, χ^2=17.636, p=<0.001, HR=0.548 (0.412-0.731)
	N2	16.8% (109)	51 (40-62)	
EMVI				
	Present	21.5% (140)	56 (46-67)	χ^2=100.946, p=<0.001, HR=3.139 (2.479-3.974)
	Absent	78.5% (510)	132 (123-141)	
Microsatellite instability				

(MSI) status				
	Intact	82.5% (536)	118 (110-127)	χ^2=2.848, p=0.091, HR=1.294 (0.957-1.750)
	Defective	14.8% (96)	98 (80-115)	
	Not assessable	2.8% (18)		
Screen Detected				
	Yes	8% (52)	120 (109-132)	χ^2=16.381, p=<0.001, HR= 3.827 (1.896-7.725)
	No	92% (598)	112 (103-120)	
Stage				
	Dukes A, (Stage 1; T1/T2, N0)	18.5% (120)	161 (145-177)	Dukes A *v* Dukes B, χ^2=5.059, p=0.025, HR=0.632 (0.422-0.947)
	Dukes B, (Stage 2; T3/T4, N0)	37.7% (245)	125 (114-135)	Dukes B *v* Dukes C, χ^2= 65.510, p=<0.001, HR=0.364 (0.281-0.470)
	Dukes C, (Stage 3; any T stage and N1 or N2)	43.8% (285)	74 (64-84)	

1. Proximal tumours are tumours proximal to the splenic flexure.
