# Supplementary material for: The Expression and Prognostic Significance of Retinoic Acid Metabolising Enzymes in Colorectal Cancer
Source: PLoS One. 2014 Mar 7;9(3):e90776. doi: 10.1371/journal.pone.0090776 (PMC3946526; doi:10.1371/journal.pone.0090776)
Supplement: Table S2 — The relationship of the expression of CYP26A1, CYP26B1 and LRAT and survival in individual Dukes stage of colorectal cancer. (PDF) [file pone.0090776.s002.pdf]

Table S2. The relationship of the expression of CYP26A1, CYP26B1 and LRAT and survival in individual Dukes stage of colorectal cancer.

		Overall		Negative v weak/moderate/strong		Negative/weak v moderate/strong		Negative/weak/moderate v strong	
		χ^2	p-value	χ^2	p-value	χ^2	p-value	χ^2	p-value
CYP26A1									
	Dukes stage								
	A	0.967	0.809	0.434	0.510	0.000	0.997	0.154	0.695
	B	2.756	0.431	0.549	0.459	0.695	0.404	0.406	0.524
	C	9.425	**0.024**	2.496	0.114	8.578	**0.003**	5.895	**0.015**
CYP26B1									
	A	3.760	0.289	2.559	0.110	0.026	0.872	0.083	0.773
	B	1.758	0.624	1.532	0.216	0.733	0.392	0.020	0.887
	C	11.127	**0.011**	2.965	0.085	10.004	**0.002**	7.215	**0.007**
LRAT									
	A	2.478	0.479	0.863	0.353	0.835	0.361	2.191	0.139
	B	1.357	0.716	0.010	0.921	0.206	0.650	0.137	0.711
	C	3.101	0.376	0.463	0.496	1.814	0.178	3.025	0.082

Significant values are highlighted in bold
