# Supplementary material for: The Expression and Prognostic Significance of Retinoic Acid Metabolising Enzymes in Colorectal Cancer
Source: PLoS One. 2014 Mar 7;9(3):e90776. doi: 10.1371/journal.pone.0090776 (PMC3946526; doi:10.1371/journal.pone.0090776)
Supplement: Table S3 — The relationship of the expression of CYP26A1, CYP26B1 and LRAT and survival in colon cancer and rectal cancer. (PDF) [file pone.0090776.s003.pdf]

Table S3. The relationship of the expression of CYP26A1, CYP26B1 and LRAT and survival in colon cancer and rectal cancer.

		Negative v weak v moderate v strong		Negative v weak/moderate/strong		Negative and weak v moderate and strong		Negative, weak and moderate *v* strong	
		χ^2	p-value	χ^2	p-value	χ^2	p-value	χ^2	p-value
CYP26A1									
	Colon	0.736	0.865	0.099	0.753	0.689	0.407	0.167	0.683
	Rectum	5.833	0.120	0.983	0.322	0.761	0.383	0.056	0.812
CYP26B1									
	Colon	8.970	0.030	3.028	0.082	8.040	0.005	5.329	0.021
	Rectum	17.129	**0.001**	4.841	**0.028**	2.209	0.137	13.780	**<0.001**
LRAT									
	Colon	3.522	0.318	1.747	0.186	3.259	0.071	2.339	0.126
	Rectum	2.750	0.432	1.103	0.294	2.597	0.107	1.883	0.170

Significant values are highlighted in bold
