# Supplementary material for: The Expression and Prognostic Significance of Retinoic Acid Metabolising Enzymes in Colorectal Cancer
Source: PLoS One. 2014 Mar 7;9(3):e90776. doi: 10.1371/journal.pone.0090776 (PMC3946526; doi:10.1371/journal.pone.0090776)
Supplement: Table S4 — The relationship of the expression of CYP26A1, CYP26B1 and LRAT and survival in proximal and distal colon cancers. (PDF) [file pone.0090776.s004.pdf]

Table S4. The relationship of the expression of CYP26A1, CYP26B1 and LRAT and survival in proximal and distal colon cancers.

		Negative v weak v moderate v strong		Negative v weak/moderate/strong		Negative/weak v moderate and strong		Negative/weak/moderate *v* strong	
		χ^2	p-value	χ^2	p-value	χ^2	p-value	χ^2	p-value
CYP26A1									
	Proximal	0.449	0.930	0.219	0.640	0.012	0.914	0.051	0.822
	Distal	1.059	0.787	0.378	0.539	0.673	0.412	0.006	0.941
CYP26B1									
	Proximal	1.728	0.631	0.076	0.783	1.280	0.258	1.285	0.257
	Distal	9.716	**0.021**	4.617	0.032	8.853	**0.003**	4.254	**0.039**
LRAT									
	Proximal	2.580	0.461	0.705	0.401	0.171	0.680	1.708	0.191
	Distal	5.484	0.140	1.329	0.249	4.227	**0.040**	0.730	0.393

Significant values are highlighted in bold
