# Supplementary material for: The Expression and Prognostic Significance of Retinoic Acid Metabolising Enzymes in Colorectal Cancer
Source: PLoS One. 2014 Mar 7;9(3):e90776. doi: 10.1371/journal.pone.0090776 (PMC3946526; doi:10.1371/journal.pone.0090776)
Supplement: Table S5 — The relationship of the expression of CYP26A1, CYP26B1 and LRAT and survival in colorectal cancers with and without EMVI. (PDF) [file pone.0090776.s005.pdf]

Table S5. The relationship of the expression of CYP26A1, CYP26B1 and LRAT and survival in colorectal cancers with and without EMVI.

		Overall		Negative v weak/moderate/strong		Negative/weak v moderate/strong		Negative/weak/moderate v strong	
		χ^2	p-value	χ^2	p-value	χ^2	p-value	χ^2	p-value
CYP26A1									
	EMVI present	4.147	0.246	<0.001	0.984	0.046	0.831	3.609	0.057
	EMVI absent	0.781	0.854	0.135	0.714	0.199	0.656	0.054	0.816
CYP26B1									
	EMVI present	2.482	0.479	0.006	0.939	1.394	0.238	1.943	0.163
	EMVI absent	7.039	0.069	4.726	**0.030**	4.235	**0.040**	3.356	0.067
LRAT									
	EMVI present	1.795	0.616	0.183	0.669	0.533	0.465	0.363	0.547
	EMVI absent	5.599	0.133	1.506	0.220	5.299	**0.021**	1.479	0.224

Significant values are highlighted in bold
