# Supplementary material for: The Expression and Prognostic Significance of Retinoic Acid Metabolising Enzymes in Colorectal Cancer
Source: PLoS One. 2014 Mar 7;9(3):e90776. doi: 10.1371/journal.pone.0090776 (PMC3946526; doi:10.1371/journal.pone.0090776)
Supplement: Table S6 — Multi-variate analysis of the whole patient cohort and MMR proficient cohort. (PDF) [file pone.0090776.s006.pdf]

Table S6.

A. Multi-variate analysis of the whole patient cohort including only paramaters that would be available if cases are considered to be a biopsy i.e. no information regarding tumour stage, nodal stage or EMVI will be available.

Variable	Wald value	p-value	Hazard ratio (95% CI)
Age	18.665	**<0.001**	1.730 (1.349-2.219)
Gender	0.098	0.754	0.963 (0.762-1.217)
Tumour site	4.365	0.113	0.855 (0.625-1.566)
Tumour differentiation	0.319	0.572	0.876 (0.553-1.387)
MSI status	1.066	0.302	1.181 (0.861-1.621)
CYP26B1	5.730	**0.017**	1.192 (1.032-1.377)
LRAT	0.011	0.917	1.015 (0.761-1.355)

Significant values are highlighted in bold

B. . Multi-variate analysis of the MSI intact patient cohort including only paramaters that would be available if cases are considered to be a biopsy i.e. no information regarding tumour stage, nodal stage or EMVI will be available.

Variable	Wald value	p-value	Hazard ratio (95% CI)
Age	18.804	**<0.001**	1.821 (1.389-2.387)
Gender	0.312	0.577	0.929 (0.719-1.202)
Tumour site	2.745	0.254	0.848 (0.613-1.523)
Tumour differentiation	0.239	0.072	0.578 (0.319-1.049)
CYP26B1	10.972	**0.001**	1.308 (1.116-1.532)
LRAT	0.197	0.657	0.930 (0.676-1.280)

Significant values are highlighted in bold
