# Supplementary material for: The Expression and Prognostic Significance of Retinoic Acid Metabolising Enzymes in Colorectal Cancer
Source: PLoS One. 2014 Mar 7;9(3):e90776. doi: 10.1371/journal.pone.0090776 (PMC3946526; doi:10.1371/journal.pone.0090776)
Supplement: Materials and Methods S1 — (PDF) [file pone.0090776.s007.pdf]

The expression and prognostic significance of retinoic acid metabolising enzymes in colorectal cancer

Supporting information

Materials and methods

Histopathological processing of colorectal cancer

The colorectal cancer excision specimens were received fresh in the diagnostic histopathology laboratory, opened along the anti-mesenteric border proximal and when appropriate distal to the tumour, washed in cold water and then fixed in 10% neutral buffered formalin for at least 48 hours at room temperature prior to further dissection and block selection. Representative tissue blocks were embedded in wax, sections were then stained with haematoxylin and eosin for histopathological diagnosis and when required tumour sections were also stained with elastic haematoxylin and eosin to permit further assessment of extramural venous invasion (EMVI). The tumours were reported according to The Royal College of Pathologists UK guidelines for the histopathological reporting of colorectal cancer resection specimens and which incorporates guidance from version 5 of the TNM staging system. The mean lymph node yield for all tumours in this study was 14.29 lymph nodes per tumour and for node negative tumours the mean lymph node yield was 15.07 (lymph node yield refers to the total number of lymph nodes retrieved from each colorectal cancer resection specimen).

Construction of colorectal cancer tissue microarray

A colorectal cancer tissue microarray was constructed containing normal colon mucosal samples (n=50), primary (n=650) and metastatic colorectal cancer samples (n=285). 99 tumours were from the period 1994-1998, 198 tumours were from 1999-2003 and 353 tumours were from the period 2004-2009. The metastases were all from tumour involved lymph nodes of the Dukes C cases. Each normal mucosal sample was acquired from at least 10 cm distant from the tumour. All the cases were reviewed and areas of tissue to be sampled were first identified and marked on the appropriate haematoxylin and eosin stained slide by an expert consultant gastro-intestinal pathologist (GIM). Two 1mm cores were then taken

from these areas of the corresponding wax embedded block using a Beecher Instruments tissue microarrayer (Sun Prairie, WI, USA) and placed in a recipient paraffin block.

Immunohistochemistry

Sections of the tissue microarray were dewaxed in xylene, rehydrated in alcohol and an antigen retrieval step performed when required [28-30]. This step consisted of microwaving the sections fully immersed in 10mM citrate buffer at pH6.0 for 20 minutes in an 800W microwave oven operated at full power. The sections were then allowed to cool to room temperature. Each primary antibody was applied as undiluted tissue culture supernatant for 60 minutes at room temperature, washed with buffer (Dako) with subsequent peroxidase blocking for 5 minutes (Dako). This was followed by a single 2 minute buffer wash after which pre-diluted peroxidase-polymer labelled goat anti-mouse/rabbit secondary antibody (Envision™, Dako) was applied for 30 minutes at room temperature, followed by further washing with buffer to remove unbound antibody. Sites of peroxidase activity were then demonstrated with diaminobenzidine as the chromogen applied for three successive 5 minute periods. Finally sections were washed in water, lightly counterstained with haematoxylin, dehydrated and mounted. Omitting the primary monoclonal antibody from the immunohistochemical procedure and replacing it with antibody diluent acted as a negative control. Normal liver which is known to express all the enzymes being studied was used as a positive control.
